# Pharmacokinetic and urinary profiling reveals the prednisolone/cortisol ratio as a valid biomarker for prednisolone administration

**DOI:** 10.1186/s12917-017-1158-5

**Published:** 2017-08-14

**Authors:** Lieven Van Meulebroek, Nathalie De Clercq, Julie Vanden Bussche, Mathias Devreese, Eric Fichant, Philippe Delahaut, Siska Croubels, Lynn Vanhaecke

**Affiliations:** 1 Faculty of Veterinary Medicine, Department of Veterinary Public Health & Food Safety, Laboratory of Chemical Analysis, Ghent University, Salisburylaan 133, 9820 Merelbeke, Belgium; 2 Faculty of Veterinary Medicine, Department of Pharmacology, Toxicology and Biochemistry, Laboratory of Pharmacology and Toxicology, Ghent University, Salisburylaan 133, 9820 Merelbeke, Belgium; 3 Département Santé, CER Groupe, Rue du Point du Jour 8, Marloie, 6900 Belgium

**Keywords:** Glucocorticoids, Prednisolone, Screening tools, Prednisolone/cortisol ratio, 20β-dihydroprednisolone, Adrenocorticotropic hormone, Urinary profiling

## Abstract

**Background:**

In Europe, synthetic corticosteroids are not allowed in animal breeding for growth-promoting purposes. Nevertheless, a high prevalence of non-compliant urine samples was recently reported for prednisolone, however, without any indication of unauthorized use. Within this context, 20β-dihydroprednisolone and the prednisolone/cortisol ratio have been suggested as potential tools to discriminate between exogenous and endogenous urinary prednisolone. In this study, the validity of these strategies was verified by investigating the plasma pharmacokinetic and urinary excretion profiles of relevant glucocorticoids in bovines, subjected to exogenous prednisolone treatment or tetracosactide hexaacetate administration to induce endogenous prednisolone formation. Bovine urine and plasma samples were analysed by liquid chromatography and mass spectrometry.

**Results:**

Based on the plasma pharmacokinetics and urinary profiles, 20β-dihydroprednisolone was confirmed as the main prednisolone-derived metabolite, being detected in the biological fluids of all 12 bovines (plasma AUC_0-inf_ of 121 h μg L^−1^ and urinary concentration > 0.695 μg L^−1^). However, this metabolite enclosed no potential as discriminative marker as no significant concentration differences were observed upon exogenous prednisolone treatment or tetracosactide hexaacetate administration under all experimental conditions. As a second marker tool, the prednisolone/cortisol ratios were assessed along the various treatments, taking into account that endogenous prednisolone formation involves the hypothalamic-pituitary-adrenal axis and is associated with an increased cortisol secretion. Significantly lower ratios were observed in case of endogenous prednisolone formation (i.e. ratios ranging from 0.00379 to 0.129) compared to the exogenous prednisolone treatment (i.e. ratios ranging from 0.0603 to 36.9). On the basis of these findings, a discriminative threshold of 0.260 was proposed, which allowed classification of urine samples according to prednisolone origin with a sensitivity of 94.2% and specificity of 99.0%.

**Conclusion:**

The prednisolone/cortisol ratio was affirmed as an expedient strategy to discriminate between endogenous and exogenous prednisolone in urine. Although the suggested threshold value was associated with high specificity and sensitivity, a large-scale study with varying experimental conditions is designated to optimize this value.

**Electronic supplementary material:**

The online version of this article (doi:10.1186/s12917-017-1158-5) contains supplementary material, which is available to authorized users.

## Background

Synthetic glucocorticoids are extensively employed in cattle for therapeutic purposes because of their well-recognized anti-inflammatory and immunosuppressive properties. Among these glucocorticoids, the most commonly used are dexamethasone methylprednisolone, and prednisolone. For example, the latter is frequently used in cattle, including dairy cows, for the treatment of allergic dermatitis, otitis, pruritus and musculoskeletal inflammation [1, 2]. Apart from the therapeutic applications, synthetic glucocorticoids may also be administered unauthorized to promote the growth of veal calves, finishing bulls and cows at the end of their production cycle [3]. Glucocorticoids tend to increase live weight gain, improve feed intake, reduce the feed conversion ratio, reduce nitrogen retention, increase the fat content and promote water retention [4]. As such, growth-promoting effects have been demonstrated for beef cattle after oral administration of prednisolone acetate (15–30 mg per animal per day) for 30–35 days [5]. Because of the strong pharmacological activity, the residues of most synthetic corticosteroids might impose a risk for food safety. Therefore, to protect consumer’s health, the administration of synthetic glucocorticoids in livestock is restricted to therapeutic applications only and should be done by a licensed veterinarian [6]. Moreover, appropriate withdrawal times have been defined for glucocorticoid treatment in order to comply with the maximum residue limits (MRLs), as they have been established for bovine edible tissues [6, 7].

In the past few years, the European Commission reported a higher prevalence of non-compliant urine samples for prednisolone [8–11], without any direct evidence of unauthorized use. Therefore, in order to account for potential endogenous prednisolone, the European Reference Laboratories have proposed a threshold level for urinary prednisolone in bovine urine of 5 μg L^−1^ [12, 13]. However, such an artificial cut-off value may still leave the possibility for false accusations or legalization of low-level prednisolone abuse. In this regard, alternatives to discriminate between exogenous and endogenous prednisolone have been suggested, i.e. usage of the prednisolone/cortisol urinary concentration ratio and analysis of 20β-dihydroprednisolone [14–17], although both have not been confirmed or validated yet. Therefore, in this study, to verify the applicability and validity of these screening tools, the pharmacokinetics and urinary excretion profiles of prednisolone, prednisone, 20α- and 20β-dihydroprednisolone were assessed during a growth-promoting and therapeutic prednisolone treatment (Additional file 1). Moreover, also a pharmacologically-induced stress treatment was considered as it has recently been evidenced that stress induction with a synthetic analogue of the adrenocorticotropic hormone (ACTH), i.e. tetracosactide hexaacetate, leads to the presence of endogenous prednisolone in bovine urine. In addition, the overall cortisol secretion during growth-promoting, therapeutic prednisolone, and ACTH treatment was assessed by profiling natural urinary glucocorticoid metabolites, i.e. cortisone, dihydrocortisone, corticosterone, deoxycorticosterone, allotetrahydrocortisol, tetrahydrocortisone, urocortisol, α-cortolone and 6β-hydroxycortisol. To date, only a few studies have been dedicated to correlate endogenous (e.g. cortisol) and exogenously administered glucocorticoids (e.g. dexamethasone, prednisolone) as a means to estimate the degree of glucocorticoid resistance or super sensitivity [18–20]. Nevertheless, a deepened knowledge on these correlations is believed to support the understanding of endogenous formation mechanisms and might yield an adequate screening tool.

## Methods

### Test animals

Twelve clinically healthy cows of a mixed breed were housed under controlled experimental conditions at the animal facilities of Centre d’Economie Rurale (CER, Marloie, Belgium). These cows were 2 to 6 years of age and had a body weight between 370 and 600 kg. They were fed a commercial diet, with ad libitum access to water and hay. During the entire study, animals were kept in three separate groups (4 animals per group), all housed in a half-covered pen. Prior to the in vivo study, an initial acclimatization period of 10 days was foreseen, allowing adaptation to the environmental and feeding conditions. This study was approved by CER’s Ethical Committee (CE/Sante/ET/004).

### Experimental protocol

**Fig. 1:**
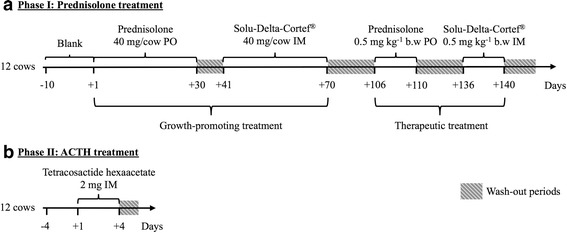
Schematic representation of the in vivo study. The various experimental treatments include oral (PO) and intramuscular (IM) prednisolone administration (**a**) and the treatment with tetracosactide hexaacetate, a synthetic analogue of ACTH (**b**). The wash-out periods are indicated in blue. This experimental procedure was implemented to each of the test animals (*n* = 12)

After acclimatization (Fig. 1A), animals were subjected to a similar oral (per os, PO) and intramuscular (IM) prednisolone treatment sequence. First, a growth-promoting treatment (long-term, 40 mg per cow a day, PO and IM) was applied, followed by a therapeutic treatment (short-term, 0.5 mg per kg bodyweight a day, PO and IM) (Fig. 1A). Then, a washout period of 11 weeks was incorporated, after which tetracosactide hexaacetate (2 mg per day) was administered intramuscularly for 4 days to mimic stress (Fig. 1B). All types of treatment were executed at 8 h in the morning.

### Phase I: Prednisolone treatments

The growth-promoting treatment started with 30 consecutive days of PO administration of 40 mg per day of prednisolone in capsules (prednisolone, Fagron, Belgium) (Day + 1 till Day + 30), followed by a washout period of 10 days. Next, IM injections of 40 mg per day of Solu-Delta-Cortef® (prednisolone sodium succinate, Zoetis, Belgium) were given for 30 consecutive days (Day + 41 till Day + 70). Before the start of the therapeutic prednisolone treatment, a washout period of 35 days was foreseen.

During the therapeutic treatment, a similar experimental set-up was implemented. First, the animals received 0.5 mg prednisolone per kg body weight PO for 5 days (Day + 106 till Day + 110), which was followed by a washout period of 25 days. Next, IM injections of 0.5 mg of Solu-Delta-Cortef® per kg body weight were given during 5 consecutive days (Day + 136 till Day + 140). Until 32 days (Day + 141 till Day + 172) after the last prednisolone administration, urine samples were collected in order to monitor the reconversion to the natural glucocorticoid body state.

### Phase II: ACTH treatment

After phase I, a washout period of 11 weeks was considered. Subsequently, all animals received IM injections of 2 mg tetracosactide hexaacetate (Utrecht University, Faculty of Veterinary Medicine, Utrecht, The Netherlands), corresponding to 200 I.U. of ACTH, during 4 consecutive days.

### Sample collection

During prednisolone treatments, urine samples were each time collected in the morning, prior to prednisolone administration. These samples were obtained by a veterinarian using a probe (to prevent faecal contamination) and were immediately portioned into 15-mL tubes, which were then stored in the dark at −80 °C until analysis. During the growth-promoting treatment, samples were collected every five days whereas during the therapeutic treatment, urine samples were collected the first, third and last day. In addition, samples were also collected every day during the acclimatization period and every five days during the washout periods. Also, urine samples were collected twice a day during the ACTH treatment period, whereby samples were collected prior to and at 4 h (Day + 1 and Day + 2) or at 6 h (Day + 3 and Day + 4) after tetracosactide hexaacetate administration.

Blood samples were collected in the morning, thereby applying a similar sampling strategy as for urine. In addition, blood samples for pharmacokinetic analysis were collected at the beginning and end of each treatment period at time 0 (just before administration), 15 min, 30 min, 45 min, 1 h, 2 h, 4 h, 6 h, 8 h, and 24 h (post-administration). With each collection, 5 mL of blood was sampled into heparin tubes. One hour after collection, blood was centrifuged at 600 x g during 15 min at 4 °C and divided into 2-mL plasma aliquots, which were then immediately stored at −20 °C until analysis.

### Reagents and chemicals

Standards of prednisolone, prednisone, cortisone, cortisol, dihydrocortisone, aldosterone, allotetrahydrocortisol, urocortisol, tetrahydrocortisone, corticosterone, deoxycorticosterone, α-cortolone and 6β-hydroxycortisol were purchased from Sigma-Aldrich (St. Louis, MO, USA). Internal standards were prednisolone-d_8_ (TRC, Canada), cortisol-d_4_ and prednisolonde-d_4_ (Sigma-Aldrich). Reagents were of analytical grade (Merck, Darmstadt, Germany) when used for extraction purposes and of liquid chromatography-mass spectrometry (LC-MS) Optima grade (Fisher Scientific, Loughborough, UK) for ultra-high performance LC (UHPLC) tandem MS (MS/MS) and high-resolution MS (HRMS) applications. Ultrapure water was obtained by usage of a purified-water system (Sartorius AG, Goettingen, Germany). Stock solutions were prepared in ethanol at a concentration of 200 μg mL^−1^ and stored in dark glass bottles at −20 °C.

### Sample preparation

#### Urine

A detailed description of the analytical procedure, which was used for glucocorticoid extraction from urine, has been given in earlier work [21]. In brief, 5 mL of urine was enriched with the internal standards cortisol-d_4_ and prednisolone-d_8_ to reach final concentration of 10 μg L^−1^. Next, a twofold liquid-liquid extraction with tert-butyl methyl ether was applied, whereby the organic phases were collected, pooled and dried under a gentle stream of nitrogen at 50 °C. The residue was then dissolved in 100 μL of initial mobile phase conditions and transferred into an LC- vial.

#### Plasma

Two mL of vortexed plasma was spiked with the internal standards cortisol-d_4_ and prednisolone-d_4_ to obtain final concentrations of 10 μg L^−1^. Glucocorticoids were extracted by liquid-liquid extraction, thereby using 5 mL acetonitrile. After 30 min of extraction, samples were centrifuged at 3760 x g for 10 min at 10 °C. Then, the supernatants were collected and evaporated under a gentle stream of nitrogen at 40 °C. The residue was suspended in 200 μL of water-acetonitrile (80/20, *v*/v) and transferred into an LC-vial.

### Mass spectrometric detection

#### UHPLC-Orbitrap MS for urine

Glucocorticoid analysis of urine was performed by UHPLC-Orbitrap MS, according to the method of De Clercq et al. (2013) [21]. Chromatographic separation of the target analytes was thereby achieved using an Accela UHPLC system (Thermo Fisher Scientific, San José, USA), equipped with a Nucleodur Isis C18 column (1.8 μm, 100 mm × 2 mm, Macherey-Nagel, Düren, Germany). High-resolution mass spectrometric analysis was performed with an Exactive™ single-stage Orbitrap mass spectrometer (Thermo Fisher Scientific), equipped with a heated electrospray ionization probe (HESI II), operating in polarity switching mode. Instrument control and data processing were carried out by Xcalibur 2.1 software (Thermo Fisher Scientific, San José, USA).

#### UHPLC-MS/MS analysis for plasma

The chromatographic analysis of glucocorticoids in plasma was performed by a Waters Acquity system (Waters, Manchester, UK) according to Delahaut et al. (2014) [16]. Chromatographic separation of the target analytes was thereby achieved using an Acquity C18 column (1.7 μm, 125 × 3 mm). Mass spectrometric analysis was performed with a Xevo TSQ tandem mass spectrometer (Waters Corporation, Manchester, UK), operating in the positive ion electrospray mode and applying multiple reaction monitoring (MRM). For each target compound, two transitions were monitored (Additional file 2), the first being the quantifier and the second being the qualifier. For quantification, two internal standards were used: prednisolone-d_4_ and cortisol-d_4_. Instrument control and data processing were carried out by MassLynx and QUANLYNX software (Waters Corporation, Manchester, UK) respectively.

A brief validation of the newly developed method for glucocorticoid analysis of plasma was performed based on Commission Decision 2002/657/EC guidelines [22]. The method performance in terms of repeatability, within-laboratory reproducibility, recovery, CC_α_ and specificity was thereby assessed. Plasma samples that were used for validation were obtained from non-medicated cows (*n* = 3), which were housed at the animal facilities of CER. Linearity was evaluated based on eight-point matrix-matched calibration curves with concentration levels ranging from 0.25 to 20 μg L^−1^ for prednisolone, prednisone, 20α-dihydroprednisolone, and 20β-dihydroprednisolone and from 0.5 to 40 μg L^−1^ for cortisol, cortisone, and dihydrocortisone. Repeatability was determined by analysis of samples that were spiked with the target compounds, thereby considering seven replicates for two different concentration levels, i.e. 0.50 and 5 μg L^−1^ for prednisolone, prednisone, 20α-dihydroprednisolone, and 20β-dihydroprednisolone and 1 and 10 μg L^−1^ for cortisol, cortisone, and dihydrocortisone. For evaluation of the within-laboratory reproducibility, the specified analyses were performed on two different occasions, with a different operator on the second occasion. The CC_α_ was derived from chromatograms and corresponded to a concentration giving a peak with a signal-to-noise ratio of 3. Specificity was evaluated by analyzing a potential interfering substance (methylprednisolone) to verify potential cross-talk during analysis.

#### Quantitation and normalization

Due to the broad concentration range expected in the urine samples during the different prednisolone treatments, quantitation of the various urinary glucocorticoid compounds was based on two eight-point calibration curves (using area ratios), which were prepared in urine matrix. Samples were thereby fortified with all glucocorticoid standards to reach concentrations from 0.50 to 75 ng mL^−1^ and from 100 to 200 ng mL^−1^ for cortisol, cortisone, dihydrocortisone, prednisolone, prednisone, methylprednisolone, 20α-dihydroprednisolone, and 20β-dihydroprednisolone. The employed urine matrix was previously verified to contain no residues of prednisolone, prednisone, 20α-dihydroprednisolone and 20β-dihydroprednisolone, but the other glucocorticoids were found to be endogenously present. Therefore, the endogenous concentration levels of cortisol, cortisone, and dihydrocortisone were determined as the average of five non-fortified urine samples and taken into account during quantitation. In addition, since urine is a matrix prone to dilution, normalization by means of the specific gravity (Pocket Refractometer™, Atago, Tokyo) was applied using the Levine-Fahy eq. [23].

#### Pharmacokinetic analysis

Pharmacokinetic analysis was performed with WinNonlin 6.3 (Pharsight Corporation, St-Louis, USA). Plasma concentration-time profiles were modeled using a one-compartmental model for PO and a two-compartmental model for IM prednisolone administration. The most important pharmacokinetic parameters were the peak plasma concentration (C_max_), time to reach the peak plasma concentration (T_max_), area under the plasma concentration-time curve from time 0 to time inf (AUC_0-inf_), absorption rate constant (k_a_), absorption half-life (T_1/2a_), apparent clearance (Cl/F) and apparent volume of distribution (Vd/F). Additionally, for two-compartmental methods, the distribution rate constant (k_elα_), the elimination rate constant (k_elβ_) and elimination half-life (T_1/2elβ_) were also determined. The coefficient of determination (R^2^) was hereby used as an indicator for the goodness-of-fit. For the main metabolites, only C_max_, T_max_, AUC_0-inf_, k_el_ en T_1/2el_ were calculated.

### Statistical analysis

All pharmacokinetic parameters were compared between administration routes according to dose (therapeutic and growth-promoting), thereby using one-way analysis of variance (ANOVA) (*p*-value ≤ 0.05) (SPSS 21, IBM, USA). Urinary concentrations were statistically evaluated using Student’s t-test and one-way ANOVA with post-hoc Tukey’s multiple comparisons test.

## Results

### Method validation

For each of the targeted compounds, the determination coefficient (R^2^) was above 0.99 for all three calibration curves, established in plasma and analyzed on three different days. The other performance characteristics of the validation are presented in Additional file 3. Recoveries ranged from 92 to 107%. Repeatability and within-laboratory reproducibility were evaluated based on the coefficients of variation (RSD) and were below the 15%-tolerance level, specified in CD 2002/657/EC [22], except for 20α-dihydroprednisolone (22.3% at 0.5 μg L^−1^), which was nevertheless considered acceptable because of the low target concentration.

### Pharmacokinetics of prednisolone and its metabolites

**Fig. 2:**
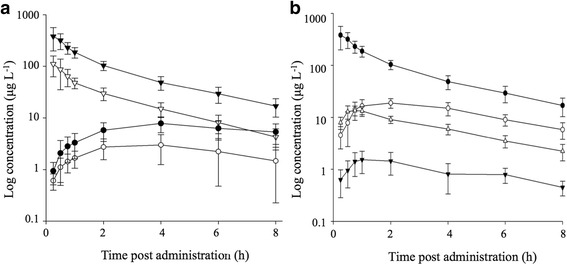
**a** Plasma concentration profile for prednisolone, as observed after growth-promoting or therapeutic prednisolone administration. Results are expressed as mean plasma concentration levels ± SD (*n* = 12). The growth-promoting and therapeutic treatment are represented by black and white symbols, respectively. Hereby, a distinction is made between oral (circle) and intramuscular (triangle) prednisolone administration. **b** Plasma concentration profile for prednisolone, 20β-dihydroprednisolone, prednisone, and 20α-dihydroprednisolone as observed after intramuscular therapeutic administration. Results are expressed as mean plasma concentration levels ± SD (*n* = 12). Prednisolone is represented by black circles, 20β-dihydroprednisolone by white circles, prednisone by white triangles and 20 α-dihydroprednisolone by black triangles

The pharmacokinetic parameters were determined for plasma prednisolone during the growth-promoting and therapeutic treatments (Fig. 2. A), thereby using a one- (PO) or two-compartmental (IM) model. A growth-promoting (40 mg day^−1^) and therapeutic (0.5 mg kg^−1^ day^−1^) PO administration of prednisolone in cattle resulted in a relatively fast absorption with C_max_ being reached within 2.95 h and 3.85 h (T_max_), respectively (Table 1). Evidently, prednisolone absorption in case of IM administration was much faster, with T_max_ ranging from 0.168 to 0.866 h. The absorption (T_1/2a_) and elimination half-life (T_1/2el_) for unbound prednisolone after PO and IM administration was considered rather independent of dose, although the highest IM dose (0.5 mg kg^−1^ day^−1^) showed a somewhat increased T_1/2el_. The Vd/F for oral administered prednisolone increased from 11.95 to 30.06 L kg^−1^ during, respectively, growth-promoting and therapeutic treatment. The same effect was noticed during the two types of IM prednisolone administration.

**Table 1:** Pharmacokinetic parameters for prednisolone after oral and intramuscular administration during growth-promoting and therapeutic treatment

	Oral treatment		Intramuscular treatment	
	40 mg day^−1^	0.5 mg kg^−1^ day^−1^	40 mg day^−1^	0.5 mg kg^−1^ day^−1^
C_max_ (μg L^−1^)	2.96 ± 1.50	7.01 ± 1.52	117 ± 25.8	156 ± 78.1
T_max_ (h)	2.95 ± 0.820	3.85 ± 0.909	0.168 ± 0.063	0.866 ± 0.273
AUC_0-tinf_ (h μg L^−1^)	26.1 ± 19.1	75.5 ± 25.0	232 ± 17.5	678 ± 11.5
k_a_ (h^−1^)	0.363 ± 0.099	0.307 ± 0.129	17.1 ± 11.2	1.93 ± 0.395
T_1/2a_ (h)	2.05 ± 0.588	2.53 ± 0.854	0.064 ± 0.052	0.370 ± 0.083
k_el_ (h^−1^)	0.362 ± 0.091	0.245 ± 0.053	0.325 ± 0.046	0.132 ± 0.092
T_1/2el_ (h)	2.04 ± 0.553	2.97 ± 0.728	2.16 ± 0.308	3.80 ± 0.499
Vd/F (L kg^−1^)	11.9 ± 4.89	30.1 ± 9.98	0.503 ± 0.215	2.47 ± 1.61
Cl/F (L h^−1^ kg^−1^)	4.66 ± 2.68	7.15 ± 1.97	0.346 ± 0.026	0.737 ± 0.012

Average parameter values and SD were determined on the basis of 12 samples. Therapeutic treatment concerned the administration of 0.5 mg prednisolone kg^−1^ day^−1^, whereas during growth-promoting treatment 40 mg day^−1^ was administered

Pharmacokinetic parameters (one-compartmental) of the main prednisolone metabolites, i.e. prednisone, 20α-, and 20β-dihydroprednisolone, were only considered for the IM therapeutic prednisolone treatment (Table 2) as plasma concentration levels were below the associated CC_α_ for all these metabolites during oral therapeutic treatment and growth-promoting treatments (PO and IM). Based on the AUC_0-inf_, it was determined that 20β-dihydroprednisolone is the most abundant prednisolone-derived metabolite in plasma. Additionally, 20β-dihydroprednisolone could be detected already 15 min after IM prednisolone administration. Maximum plasma concentration levels for this metabolite were reached about 2 h after start of the treatment (Fig. 2. B). For prednisone, a similar T_max_ was observed as for prednisolone (0.857 h and 0.866 h, respectively), indicating a very fast conversion of prednisolone into prednisone by the 11β-hydroxysteroid dehydrogenase type 2 enzyme in the liver [24].

**Table 2:** Pharmacokinetic parameters for prednisone, 20α-dihydroprednisolone and 20β-dihydroprednisolone after intramuscular administration during therapeutic prednisolone treatment

	Prednisone	20α-dihydroprednisolone	20β-dihydroprednisolone
C_max_ (μg L^−1^)	13.3 ± 2.56	1.64 ± 0.677	19.2 ± 4.31
T_max_ (h)	0.857 ± 0.136	1.42 ± 0.413	2.17 ± 0.571
AUC_0-inf_ (h μg L^−1^)	58.5 ± 12.1	7.07 ± 3.33	121 ± 26.9
k_el_ (h^−1^)	0.307 ± 0.101	0.553 ± 0.184	0.349 ± 0.086
T_1/2el_ (h)	2.43 ± 0.649	1.53 ± 1.02	2.09 ± 0.522

Average parameter values and SD were determined on the basis of 12 samples. Intramuscular therapeutic treatment concerned the injection of 0.5 mg prednisolone kg^−1^ day^−1^

During tetracosactide hexaacetate administration, traces of prednisolone in plasma could be detected, but were below CC_α_. As reliable quantification is hereby not possible, pharmacokinetic parameters were not determined.

### Urinary excretion profile of prednisolone and its metabolites

#### Growth-promoting treatment

Based on the analysis of the urine samples that were collected prior to prednisolone administration, it was verified that prednisolone, prednisone, 20α-, and 20β-dihydroprednisolone were not endogenously present at detectable concentration levels. After five days of oral prednisolone treatment (40 mg day^−1^), urinary prednisolone reached an average concentration of 0.832 ± 0.38 μg L^−1^. When animals were given a same dose by intramuscular injection, an average prednisolone concentration of 1.16 ± 1.08 μg L^−1^ was reached. These urinary prednisolone concentrations are below the threshold of 5 μg L^−1^, suggested by the EURL. During the following 25 days, the urinary prednisolone concentrations remained rather constant for both treatments. Eventually, 24 h after the growth-promoting treatment was ended, prednisolone concentrations started to decrease. Undetectable levels were reached after 5 days, which appeared to be independent of the administration route.

During growth-promoting treatment, prednisolone and 20β-dihydroprednisolone were detected in all urine samples, whereas 20α-dihydroprednisolone was only present in the urine of 8 out of 12 animals (Table 3. A). Prednisone was not detected in any urine sample when prednisolone was administered orally, being in contrast with intramuscular injection where prednisone was detected in the urine samples from 4 animals. The major prednisolone metabolite, i.e. 20β-dihydroprednisolone, was found until 24 h after the end of the IM treatment, with undetectable levels after five days.

**Table 3:** Urinary prednisolone, prednisone, 20α-dihydroprednisolone and 20β-dihydroprednisolone concentrations during growth-promoting and therapeutic prednisolone treatment

	Prednisolone	Prednisone	20α-dihydroprednisolone	20β-dihydroprednisolone
A1. Oral growth-promoting treatment				
N° positive	12	0	8	12
C_min_ (μg L^−1^)	0.027	-	0.153	0.695
C_max_ (μg L^−1^)	1.17	-	3.41	14.10
Mean (μg L^−1^)	0.832	-	0.771	4.35
SD (μg L^−1^)	0.381	-	0.544	2.44
A2. Intramuscular growth-promoting treatment				
N° positive	12	4	8	12
C_min_ (μg L^−1^)	0.014	0.030	0.138	0.938
C_max_ (μg L^−1^)	8.44	2.17	1.79	14.2
Mean (μg L^−1^)	1.16	0.311	0.472	4.91
SD (μg L^−1^)	1.08	0.413	0.308	3.15
B1. Oral therapeutic treatment				
N° positive	12	2	8	12
C_min_ (μg L^−1^)	0.038	0.033	0.156	1.07
C_max_ (μg L^−1^)	1.36	0.306	2.85	15.15
Mean (μg L^−1^)	0.922	0.125	0.552	5.13
SD (μg L^−1^)	0.428	0.108	0.635	1.20
B2. Intramuscular therapeutic treatment				
N° positive	12	12	12	12
C_min_ (μg L^−1^)	0.528	1.08	3.32	8.97
C_max_ (μg L^−1^)	189	22.5	127	100
Mean (μg L^−1^)	31.3	8.17	14.9	37.9
SD (μg L^−1^)	40.7	5.91	26.0	25.0

For each of the treatment set-ups (A1, A2, B1, B2), 12 bovines were considered. In case that a glucocorticoid was not found in the urine samples of all animals, means ± SD were determined based on the positive urine samples only

#### Therapeutic treatment

After three days of PO and IM therapeutic administration of prednisolone, mean urinary prednisolone concentrations of respectively 0.922 μg L^−1^ and 20.1 μg L^−1^ were retrieved. After two more days of IM injection, the urinary average prednisolone concentration further increased to 42.4 μg L^−1^, whereas the concentration remained rather constant in case of oral treatment. After ending the PO treatment, the urinary excretion profile of prednisolone revealed a very strong decrease. Indeed, within 48 h after final administration, prednisolone concentration levels were diminished with almost 80%. Eventually, after 5 days, no more prednisolone could be detected at concentration levels above the CC_α_. For IM injection, prednisolone concentrations above the CC_α_ were observed in two animals up to 10 days after final treatment.

During PO administration of prednisolone, all urine samples contained prednisolone and 20β-dihydroprednisolone, whereas prednisone and 20α-dihydroprednisolone could only be detected in 2 and 8 animals, respectively. For IM prednisolone injection, all target glucocorticoids were detected in the urine samples of all animals (Table 3B). At the end of the therapeutic treatment, 20β-dihydroprednisolone could be detected in urine up to 24 h and 5 days for PO and IM prednisolone treatment, respectively. The other metabolites were not found after ending PO administration and only until 24 h after ending the IM treatment.

#### ACTH treatment

The synthesis of various glucocorticoids may be induced by treatment with tetracosactide hexaacetate, affecting the hypothalamic-pituitary-adrenal axis. In this study, detectable concentration levels of prednisolone, prednisone, 20β-dihydroprednisolone and 20α-dihydroprednisolone were observed under this pharmacologically-induced stress. Hereby, concentration levels for prednisolone in urine ranged from 0.120 to 6.45 μg L^−1^ (average 1.45 μg L^−1^), 4 h after tetracosactide hexaacetate administration (Day + 1 and Day + 2). After 6 h, prednisolone concentration levels were significantly lower (*p*-value ≤ 0.05), although still detectable with a concentration range from 0.169 to 0.729 μg L^−1^ (average 0.318 μg L^−1^). The metabolites prednisone, 20α-, and 20β-dihydroprednisolone were also detected, 4 h after tetracosactide hexaacetate administration (Day + 1 and Day + 2), showing concentrations that ranged from 0.697 to 14.4 μg L^−1^ (average 3.51 μg L^−1^), 3.31 to 19.1 μg L^−1^ (average 6.31 μg L^−1^), and 0.407 to 33.2 μg L^−1^ (average 8.12 μg L^−1^), respectively. At 6 h after treatment (Day + 3 and Day + 4), the metabolite concentrations were 2 to 3 times lower, but still present at detectable levels in the urine from all animals. Eventually, 24 h after the last tetracosactide hexaacetate administration, no residues of prednisolone or its metabolites could be detected.

**Fig. 3:**
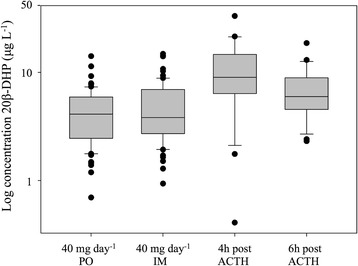
Urinary concentration profile of 20β-dihydroprednisolone during growth-promoting and ACTH administration. Concentrations of 20β-dihydroprednisolone (20β-DHP) are log-transformed and presented for the oral (PO1) and intramuscular (IM1) growth-promoting treatment, as well as for the ACTH treatment (with sampling moments at 4 h and 6 h after administration)

In this study, urinary 20β-dihydroprednisolone was thus detected upon growth-promoting (PO and IM) prednisolone treatment as well as in the pharmacologically-induced stress situation (Fig. 3). Hereby, no significant differences (*p* ≥ 0.05) were observed between the 20β-dihydroprednisolone concentrations after PO or IM prednisolone administration and those determined at 6 h post-ACTH treatment. This is not the case when the urinary concentrations are considered 4 h after administration of tetracosactide hexaacetate, showing a significant difference (*p* ≤ 0.05).

### Urinary excretion profile of natural glucocorticoids

#### Acclimatization

During the acclimatization period, the three most abundant steroids in urine were cortisol (ranging from 0.411 to 4.26 μg L^−1^, average 1.79 μg L^−1^), cortisone (ranging from 0.472 to 5.53 μg L^−1^, average 2.68 μg L^−1^) and dihydrocortisone (ranging from 0.222 to 9.36 μg L^−1^, average 2.27 μg L^−1^). Other steroids and associated metabolites were detected at significantly lower concentrations.

#### Growth-promoting and therapeutic treatment

**Fig. 4:**
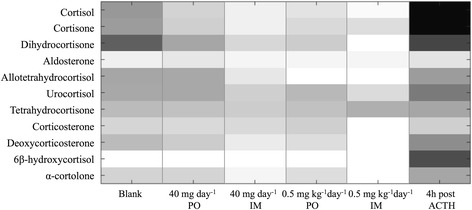
Heat map with the relative abundance of urinary steroids and metabolites along in vivo treatments. Within this heat map, urinary relative abundances are presented for the acclimatization period (Blank), growth-promoting treatment (PO1 and IM1), therapeutic treatment (PO2 and IM2), and ACTH treatment. The intensities of each ion were averaged (*n* = 12) and then log-transformed. Shades of black and white represent, respectively, higher and lower intensities as observed for the different treatments

The relative intensity changes of the target natural metabolites during the various treatments are visualized by a heat map (Fig. 4). It was hereby noted that the intensity of most metabolites showed a steady decrease when prednisolone dose increased.

When a growth-promoting dose of prednisolone was administered, regardless of the administration route, a significant decrease (*p*-value ≤ 0.05) of the urinary concentration was observed for cortisol, cortisone, dihydrocortisone, and deoxycorticosterone (i.e. compared to Blank). After ending the growth-promoting treatment, it took 5 days before cortisol and its associated metabolites reached their basal concentration levels again.

During therapeutic administration, a 2- to 5-fold intensity decrease for the urinary metabolites was noticed for the oral treatment, whereas during IM therapeutic treatment, almost no residues of the glucocorticoids dihydrocortisone, allotetrahydrocortisol, corticosterone, deoxycorticosterone, 6β-hydrocycortisol and α-cortolone remained (*p*-values ≤ 0.05, i.e. compared to Blank). It took 12 days after ending the therapeutic treatment, before cortisol and its metabolites reached their basal concentration levels again.

#### ACTH treatment

Urinary glucocorticoid profiles were evaluated during the first 2 days of ACTH treatment. It was thereby concluded that treatment with tetracosactide hexaacetate resulted in significantly (*p*-value ≤ 0.05) increased urinary secretion of cortisol, cortisone, dihydrocortisone, tetrahydrocortisol, 6β-hydrocycortisol and α-cortolone (Fig. 4). The most intense increases were noticed for cortisol (70-fold), cortisone (40-fold), and 6β-hydrocycortisol (35-fold). In addition, the mineralocorticoid aldosterone was evaluated as well [25], but showed only a 1.5-fold increase, which is much less than the observed 70-fold increase of cortisol.

## Discussion

In this study, it was aimed to verify the validity of the prednisolone/cortisol ratio and capacity of 20β-dihydroprednisolone to discriminate between endogenous and exogenous urinary prednisolone [16, 19]. Indeed, there is a strong need for an adequate screening and confirmation tool that is able to unambiguously affirm the origin of detected prednisolone as the EURL suggested discriminative threshold value of 5 μg L^−1^ [12, 13] is not fully conclusive. This was also evidenced in this study as urinary prednisolone concentrations were frequently below this threshold during both growth-promoting and therapeutic treatment. In this context, the above-mentioned screening strategies were comprehensively assessed by evaluating the urinary profiles and plasma pharmacokinetics of relevant glucocorticoids, as being measured during in vivo experiments, which intended to mimic situations of endogenous prednisolone formation and dose-variant exogenous prednisolone administration.

Based on the plasma pharmacokinetics and urinary profiles, it was concluded that 20β-dihydroprednisolone is the most abundant prednisolone-derived metabolite in both matrix types, independently of administration route. These results are in line with the findings of Nebbia et al. (2014) [3], who acknowledged 20β-dihydroprednisolone as the main urinary metabolite of prednisolone in therapeutically treated bovines (*n* = 14). As such, based on the abundant presence of 20β-dihydroprednisolone, this metabolite was indeed considered interesting to be tested as a potential urinary discriminative marker. In the prednisolone-treated animals, this metabolite could be detected in all urine samples, independently of the prednisolone administration route or dose. However, 20β-dihydroprednisolone was also detected in all samples from the ACTH treatment, which intended to induce endogenous formation of prednisolone. Hereby, no significant differences (*p*-value > 0.05) could be revealed between the urinary concentrations for the growth-promoting treatment and those in the samples that were collected 6 h after tetracosactide hexaacetate administration. On the other hand, at 4 h after tetracosactide hexaacetate treatment, urinary concentrations of 20β-dihydroprednisolone were noted to be significantly higher (*p*-value < 0.05) than those obtained during the growth-promoting treatment. Nevertheless, it is stated that 20β-dihydroprednisolone is no adequate marker metabolite as its discriminative potential will be strongly dependent on the time of sampling, relative to the triggering (stress) moment of endogenous prednisolone formation, whereby a small shift in time may already yield a completely different decision with respect to the origin of detected prednisolone. Moreover, it would be very difficult or even impossible to set a discriminative threshold for 20β-dihydroprednisolone as a large inter-individual variation in urinary concentration was noted for this metabolite. In this regard, it can be stated that metabolic processes and their sensitivity to specific triggers are strongly depending on the individual animal, which was also concluded when considering the urinary profiles of 20α-dihydroprednisolone within the prednisolone treatments. It was hereby observed that for both the oral growth-promoting and therapeutic treatment, the same 8 out of 12 animals showed detectable concentration levels of 20α-dihydroprednisolone in their urine. As this was verified to be independent of body weight or age, individual differences in metabolism are assumed to be responsible for this.

**Fig. 5:**
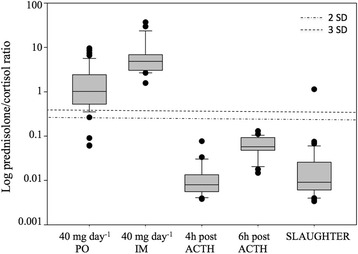
Boxplot for urinary prednisolone/cortisol ratios as determined for growth-promoting, ACTH treatment and at slaughter. Urinary concentration ratios were log-transformed. Within the growth-promoting treatment, a distinction between oral (PO1) and intramuscular (IM) prednisolone treatment was made

**Fig. 6:**
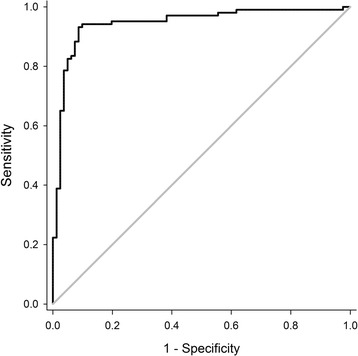
ROC-curve for sensitivity and specificity in determining the origin of prednisolone using the prednisolone/cortisol ratio. Data for natural stress (slaughter, *n* = 36), pharmacologically induced stress (ACTH, *n* = 45), and exogenously administered prednisolone (PO and IM, *n* = 103) were used for establishing the ROC-curve. The diagonal line represents the class discrimination on random chance with an AUC of 0.5 whereas a perfect discrimination is represented by an AUC of 1. The AUC of this particular ROC curve was 0.945

Evaluation of the prednisolone/cortisol ratio was conducted by profiling the natural glucocorticoid metabolites during dose-variant prednisolone treatments (growth-promoting and therapeutic) as well as induced formation of endogenous prednisolone. For the prednisolone treatments, a dose-dependent decrease in relative intensities was observed for the natural urinary glucocorticoids, including cortisol. This indicates a significant effect of prednisolone administration on the regulation of the synthesis of endogenous glucocorticoids, being the result of a suppressed hypothalamic-pituitary-adrenal axis. Hereby, prednisolone inhibits the transcription of corticotropin-releasing hormone (CRH), which also negatively affects the pro-opiomelanocortin (POMC) transcription. The associated POMC polypeptide is a precursor for ACTH, which makes that administration of prednisolone indirectly leads to the inhibition of ACTH and eventually to a decreased production of cortisol [26]. The impact of prednisolone is hereby influenced by both the dosage and administration route [27], which has been observed in this study as well. Focusing on the growth-promoting treatment, prednisolone/cortisol ratios ranged from 0.0603 to 9.55 during PO administration and from 1.57 to 36.9 during IM administration (Fig. 5). Metabolism of cortisol was also monitored during pharmacologically induced stress, triggering the hypothalamic-pituitary-adrenal axis and associated cortisol secretion. As expected, in comparison with non-stressed conditions (i.e. Blank), a significantly higher (*p* < 0.05) urinary cortisol concentration was observed upon tetracosactide hexaacetate administration. For calculation of the associated prednisolone/cortisol ratios, it was opted to also incorporate the data from a recent study of De Clercq et al. (2015) [28], considering a situation of ‘natural’ stress (at slaughter). As such, concentration ratios ranged from 0.00379 to 0.0763 at 4 h after ACTH treatment, from 0.0147 to 0.129 at 6 h after ACTH treatment, and from 0.00315 to 1.044 awaiting slaughter (Fig. 5). Based on the increased cortisol secretion during (induced) stress, prednisolone/cortisol ratios appeared to be significantly lower (*p*-value < 0.05) than during growth-promoting prednisolone treatment. However, it is here also noted that ratios tend to change rapidly with the time relative to the moment of stress. Nevertheless, this ratio shows potential to affirm the origin of detected prednisolone, and for this, a discriminative threshold was determined. To this end, a receiver operating characteristics (ROC) curve was established (Fig. 6) (SPSS Statistics 24, BMI, USA), which described the sensitivity and specificity when deciding on the origin of detected prednisolone. Hereby, the individual prednisolone/cortisol ratios associated with natural stress (at slaughter, *n* = 36), induced stress (ACTH treatment, *n* = 45), and prednisolone treatments (PO and IM, *n* = 103) were used. It should be noted that only those samples with detectable prednisolone concentrations were taken into account and that in case of cortisol concentrations below the LOD, the ratio was set as the maximum of the concerned experimental condition. The latter was the case for some samples from PO and IM, and this for both therapeutic and growth-promoting treatment. Based on the area under the curve (AUC), which is in fact a summary measure of the ROC-curve, excellent discrimination using the prednisolone/cortisol ratio was concluded as the AUC was 0.945 (*p*-value of 4.11 e^−5^) and thus close to 1 [29]. Searching the balance between an optimal sensitivity and specificity, a threshold of 0.260 was decided, which was accompanied by a sensitivity of 94.2% and a specificity of 99.0%. Further studies should confirm the validity of this threshold in discriminating endogenous from exogenous prednisolone.

## Conclusions

This study yielded an expedient strategy to appoint the origin of detected urinary prednisolone in cattle. More specifically, the urinary prednisolone/cortisol ratio was found to specifically discriminate between exogenous and endogenous prednisolone, being the result of exogenous prednisolone administration and pharmacologically induced or natural stress, respectively. Hereby, a discriminative threshold value was proposed, i.e. a concentration ratio of 0.260, which rendered a sensitivity of 94.2% and specificity of 99.0%. As such, the prednisolone/cortisol ratio is believed to hold significant value to contribute towards a more righteous decision-making when monitoring for unauthorized usage of prednisolone in bovine breeding. However, it is acknowledged that the validity of the suggested threshold should be verified under varying conditions and on a larger scale before implementation of the prednisolone/cortisol ratio becomes possible. Alternatively, the discriminating potential of 20β-dihydroprednisolone was assessed as well, whereby no unambiguously significant concentration differences were noted between exogenous prednisolone administration and pharmacologically induced endogenous formation of prednisolone. Therefore, at this this point, 20β-dihydroprednisolone was not assigned any marker potential, although situations of prednisolone formation under natural stress should be considered before the marker potential of this metabolite can be disclaimed definitely. In this context and in contrast with the applied targeted approaches, an untargeted fingerprinting of the urinary metabolome may still be opportune to reveal (unknown) metabolite markers in order to transcend the sensitivity and specificity values, as currently linked with the prednisolone/cortisol ratio.

## Additional files

Additional file 1: Biotransformation pathway of prednisolone into prednisone, 20α-dihydroprednisolone and 20β-dihydroprednisolone by hydroxysteroid dehydrogenases. Schematic representation of the enzymatic mechanisms, underlying the formation of the major prednisolone-related metabolites. Involved enzymes concern various hydroxysteroid dehydrogenases (HSD). (TIFF 10482 kb)

Additional file 2: MS/MS-parameters for the target glucocorticoids and internal standards. Table that contains data on the selected precursor and product ions, which were selected for each of the targeted glucocorticoids. In addition, optimal parameter settings for tandem fragmentation are presented. (PDF 59 kb)

Additional file 3: Performance characteristics of the method for glucocorticoid analysis in bovine plasma. Quantitative data for the performance criteria, obtained by method validation according to CD 2002/657/EC. Whereas recovery and repeatability were evaluated on the basis of 7 samples per nominal concentration, within-laboratory reproducibility was determined by considering 14 samples. RSD represent the relative standard deviation. (PDF 66 kb)

## Abbreviations

11β-HSD2: 11β-hydroxysteroid dehydrogenase type 2
ACTH: Adrenocorticotropic hormone
AUC_0-inf_: Area under the plasma concentration-time curve from time 0 to time inf
CER: Centre d’Economie Rurale
Cl/F: Apparent clearance
C_max_: Peak plasma concentration
HESI: Heated electrospray ionization
IM: Intramuscular
k_a_: Absorption rate constant
k_elα_: Distribution rate constant
k_elβ_: Elimination rate constant
MRL: Maximum residue limit
MRM: Multiple reaction monitoring
PO: Per os
R^2^: Determination coefficient
ROC: Receiver operating characteristic
RSD: Coefficients of variation
T_1/2a_: Absorption half-life
T_1/2elβ_: Elimination half-life
T_max_: Time to reach the peak plasma concentration
UHPLC: Ultra-high performance liquid chromatography
Vd/F: Apparent volume of distribution
