# Supplementary material for: Pharmacokinetic and urinary profiling reveals the prednisolone/cortisol ratio as a valid biomarker for prednisolone administration
Source: BMC Vet Res. 2017 Aug 14;13:236. doi: 10.1186/s12917-017-1158-5 (PMC5557569; doi:10.1186/s12917-017-1158-5)
Supplement: Supplementary file 2 — MS/MS-parameters for the target glucocorticoids and internal standards. Table that contains data on the selected precursor and product ions, which were selected for each of the targeted glucocorticoids. In addition, optimal parameter settings for tandem fragmentation are presented. (PDF 59 kb) [file 12917_2017_1158_MOESM2_ESM.pdf]

Additional file 2. MS/MS-parameters for the target glucocorticoids and internal standards.

**Compound**	**Precursor ion (*m/z*)**	**Product ion (*m/z*)**	**Cone voltage (V)**	**Collision energy (eV)**
Prednisolone	361.2	147.0	20	24
		343.0	20	10
Cortisol	363.1	120.8	30	30
		309.0	30	15
Prednisone	359.3	313.3	20	10
		295.0	20	12
Cortisone	361.2	163.0	30	22
		120.9	30	30
Dihydrocortisone	363.2	163.2	36	20
		105.2	36	40
20β-dihydroprednisolone	363.1	267.1	30	15
		171.1	30	20
20α-dihydroprednisolone	363.1	267.1	30	15
		171.1	30	20
Prednisolone-d_4	365.3	347.3	20	10
Cortisol-d_4	367.2	312.2	30	15
