# Supplementary material for: Pharmacokinetic and urinary profiling reveals the prednisolone/cortisol ratio as a valid biomarker for prednisolone administration
Source: BMC Vet Res. 2017 Aug 14;13:236. doi: 10.1186/s12917-017-1158-5 (PMC5557569; doi:10.1186/s12917-017-1158-5)
Supplement: Supplementary file 3 — Performance characteristics of the method for glucocorticoid analysis in bovine plasma. Quantitative data for the performance criteria, obtained by method validation according to CD 2002/657/EC. Whereas recovery and repeatability were evaluated on the basis of 7 samples per nominal concentration, within-laboratory reproducibility was determined by considering 14 samples. RSD represent the relative standard deviation. (PDF 66 kb) [file 12917_2017_1158_MOESM3_ESM.pdf]

Additional file 3. Performance characteristics of the method for glucocorticoid analysis in bovine plasma.

**Compound**	**CC_{α} ($\mu g\ L^{-1}$)**	**Nominal conc. ($\mu g\ L^{-1}$)**	**Recovery (%)**	**Precision** Repeatability RSD (%) (n = 7)	**Precision** Within-lab reprod. RSD (%) (n = 14)
Dihydrocortisone	0.360	1	106.9	13.3	13.1
		10	104.1	3.9	5.6
Cortisol	0.107	1	105.2	13.2	14.3
		10	100.8	3.8	6.3
Cortisone	0.149	1	88.0	9.3	14.7
		10	92.7	6.5	9.5
Prednisolone	0.093	0.5	97.6	4.1	9.9
		5	101.0	3.7	4.4
Prednisone	0.075	0.5	101.6	10.2	13.5
		5	102.7	4.4	12.0
20α-dihydroprednisolone	0.047	0.5	92.7	5.2	22.3
		5	96.4	3.4	12.8
20β-dihydroprednisolone	0.095	0.5	99.1	8.7	14.5
		5	105.4	5.8	10.6
